# *TSPAN1, TMPRSS4, SDR16C5*, and *CTSE* as Novel Panel for Pancreatic Cancer: A Bioinformatics Analysis and Experiments Validation

**DOI:** 10.3389/fimmu.2021.649551

**Published:** 2021-03-18

**Authors:** Hua Ye, Tiandong Li, Hua Wang, Jinyu Wu, Chuncheng Yi, Jianxiang Shi, Peng Wang, Chunhua Song, Liping Dai, Guozhong Jiang, Yuxin Huang, Yongwei Yu, Jitian Li

**Affiliations:** ^1^College of Public Health, Zhengzhou University, Zhengzhou, China; ^2^Laboratory of Molecular Biology, Henan Luoyang Orthopedic Hospital (Henan Provincial Orthopedic Hospital), Zhengzhou, China; ^3^Henan Key Laboratory of Tumor Epidemiology, Zhengzhou, China; ^4^Henan Institute of Medical and Pharmaceutical Sciences, Zhengzhou University, Zhengzhou, China; ^5^Deparment of Pathology, First Affiliated Hospital of Zhengzhou University, Zhengzhou, China; ^6^Program in Public Health, University of California, Irvine, Irvine, CA, United States; ^7^Department of Pathology, Second Military Medical University, Shanghai, China

**Keywords:** pancreatic cancer, WGCNA, diagnostic model, machine learning, bioinformatics, panel

## Abstract

Pancreatic cancer is a lethal malignancy with a poor prognosis. This study aims to identify pancreatic cancer-related genes and develop a robust diagnostic model to detect this disease. Weighted gene co-expression network analysis (WGCNA) was used to determine potential hub genes for pancreatic cancer. Their mRNA and protein expression levels were validated through reverse transcription PCR (RT-PCR) and immunohistochemical (IHC). Diagnostic models were developed by eight machine learning algorithms and ten-fold cross-validation. Four hub genes (*TSPAN1, TMPRSS4, SDR16C5*, and *CTSE*) were identified based on bioinformatics. RT-PCR showed that the four hub genes were expressed at medium to high levels, IHC revealed that their protein expression levels were higher in pancreatic cancer tissues. For the panel of these four genes, eight models performed with 0.87–0.92 area under the curve value (AUC), 0.91–0.94 sensitivity, and 0.84–0.86 specificity in the validation cohort. In the external validation set, these models also showed good performance (0.86–0.98 AUC, 0.84–1.00 sensitivity, and 0.86–1.00 specificity). In conclusion, this study has identified four hub genes that might be closely related to pancreatic cancer: *TSPAN1, TMPRSS4, SDR16C5*, and *CTSE*. Four-gene panels might provide a theoretical basis for the diagnosis of pancreatic cancer.

## Introduction

Pancreatic cancer is the seventh leading cause of cancer-related deaths worldwide, and the mortality rate closely parallels the incidence (1). In recent years, deaths associated with pancreatic cancer are gradually increasing and it is predicted to be the second leading cause of cancer-related death by 2030 (2). In the United States, it is estimated that there will be approximately 56,770 new pancreatic cancer cases diagnosed, and 45,750 estimated deaths occurring among these new cases (3). From 2003 to 2015 statistics from China show that the age-standardized 5-year relative survival rate for pancreatic cancer was only 7.2%(4). Despite advances in pancreatic cancer treatment strategies, the prognosis remains poor, largely due to the lack of early diagnostic approaches (5). Additionally, carbohydrate antigen 19–9 is widely used for the diagnosis of pancreatic cancer, but its sensitivity and specificity are only 0.80 (95% CI: 0.77-0.82) and 0.80 (95% CI: 0.77-0.82), respectively (6, 7). Therefore, the identification of new biomarkers or a panel with high specificity and sensitivity for diagnosing pancreatic cancer are important.

In recent years, with the development of microarray and high-throughput sequencing technologies, gene expression profiles have become an effective source of biomarkers discovery. Weighted gene expression network analysis (WGCNA) has been widely used to reveal the phenotype-related genes by constructing scale-free gene co-expression networks, especially in cancers, including lung (8), bladder (9), breast (10), and pancreatic cancer (11). In developing prediction models, satisfying the sensitivity and specificity requirements are the most interesting and challenging tasks for tumor biomarker screening. Previous studies have shown that machine learning method can improve the accuracy of disease diagnosis or prognosis (12, 13), and cancer models with higher accuracy have been developed by applying those methods (14–17).

Therefore, this study was designed to explore novel biomarkers with high performance using bioinformatics. Potential genes, screened by bioinformatics, will be validated using RT-PCR and IHC experiments. Diagnostic models will be constructed using different machine learning methods and ten-fold cross-validation.

## Materials and Methods

### Data Collection and Preprocessing

**Figure 1:**
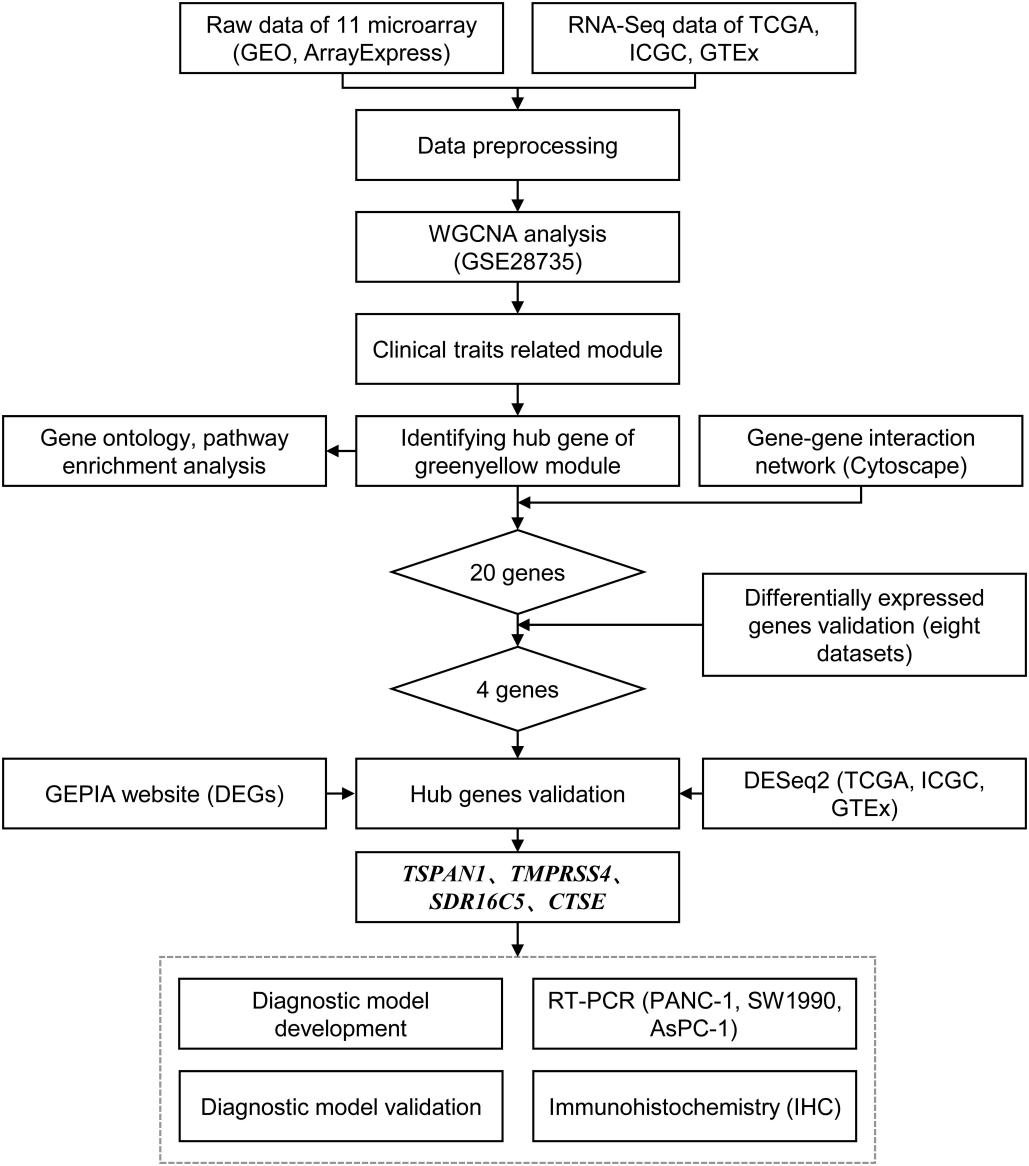
Flow chart of data preparing, analysis, validation, and model development.

The study design is shown in Figure 1. A systematic search on two electronic databases (Gene Expression Omnibus and ArrayExpress) was performed for potential datasets before 1 June 2019. Datasets with a sample size >20 were included. Eleven pancreatic cancer microarray datasets from three platforms were downloaded (Affymetrix Human Genome U133 Plus 2.0 Array, Affymetrix Human Gene 1.0 ST Array, and Affymetrix Human Genome U219 Array). The raw data were pre-processed with the “oligo” package and the “affy” packages. The Robust Multichip Average (RMA) function was used for background correction and normalization. In this study, GSE28735 was used to construct a weighted gene co-expression network because it contained the most balanced case and control samples, nine datasets (E-MEXP-2780, GSE15471, GSE16515, GSE32688, GSE71989, GSE106189, GSE62452, E-MTAB-6134, GSE62165) were combined to develop diagnostic models with a total of 818 samples, and the GSE32676 dataset with 32 samples was chosen to externally validate the model's performance. The ComBat algorithm was used to adjust the expression data from nine datasets for batch effects using the “sva” package (18). The characteristics of all microarray datasets are summarized in Supplementary Table 1.

Besides, TCGA data on RNA-sequencing (RNA-Seq) was downloaded using the “TCGAbiolinks” package (19), the ICGC data on RNA-Seq was download from Data Portal (https://dcc.icgc.org/releases/current/Projects), normal sample data was download from GTEx Portal (https://gtexportal.org/home/datasets). In total, RNA-Seq data were collected from 598 samples, including 270 cases of carcinoma and 328 cases of normal tissue.

### Weighted Gene Co-expression Network Analysis

The theoretical framework of the WGCNA algorithm has been described (20). The “WGCNA” package was used to construct the co-expression network (21). Firstly, the quality of samples and genes was checked. Then, outlier samples were removed by cluster analysis using the average linkage method. When constructing a weight co-expression network, the soft threshold power is an important parameter that can emphasize strong and reduce weak correlations between genes. The power of β = 8 (scale-free R^2^ = 0.86) was selected to ensure a scale-free network. Then, the adjacency was transformed into a topological overlap matrix (TOM), and the topological overlap dissimilarity (1-TOM) was used as hierarchical clustering input. Next, gene modules were identified using a dynamic hybrid branch cutting method with a minimum size of 30 for the gene dendrogram (22), and gene modules with a height of <0.25 were combined. An important goal of WGCNA is to detect the gene module subsets that are closely related to clinical traits. Genes within an identified module may have great biological significance. To this end, gene significance (GS) and module significance (MS) were calculated. Also, module membership (MM) was defined to select highly corrected modules with certain clinical traits.

### Identification of the Hub Genes

In gene networks, genes that have many interactions with other genes are defined as hub genes. Hub genes usually play an important role in a biological system (23). All genes in the significant module were included to construct a gene-gene interaction network using the “cytoHubba” Cytoscape plugin (24, 25). The top 20 genes, ranked by degrees of interactions, were selected. These genes may play important roles in the pathogenesis of pancreatic cancer. Then, differentially expressed genes (DEGs) were identified for GSE15471, GSE28735, GSE62165, GSE32688, GSE71989, GSE62452, GSE62165, and GSE32676 datasets, respectively. The “limma” (26) package was used to identify DEGs, false discovery rate (FDR) <0.05 and |log2 fold change (FC)| > 1 were set as the cut-offs. Overall, the hub genes were determined by the intersection of the top 20 genes and the results of the eight DEGs analyses.

### Validation of the Hub Genes

To validate hub gene expression in pancreatic cancer and normal tissues, the GEPIA tool (http://gepia.cancer-pku.cn/) was firstly applied using the RNA-Seq data (27). It is worth emphasizing that the GEPIA website included the TCGA and GTEx datasets (19, 28). And the transcripts per million (TPM) algorithm was used to measure RNA expression (29). Using the “DESeq2” package, further validation was performed based on the negative binomial distribution model using the raw counts of TCGA, ICGC, and GTEx data (30).

### Reverse Transcription PCR (RT-PCR)

cDNA was synthesized using 1 μg of total RNA isolated from three pancreatic cancer cell lines (PANC-1, GCC-PA0001RT; SW1990, GCC-PA0004RT; and AsPC-1, GCC-PA0006RT) and RT-PCR was performed using 400 ng cDNA per 12 μl reactions. The primer sequences used in RT-PCR are described in Table 1. Relative expression abundance was determined by ΔCt=Ct (hub gene)—(*GAPDH*). ΔCt≦12, 12 < ΔCt <16 and ΔCt≧16 were considered to be a high expression abundance, moderate expression abundance and low expression abundance, respectively.

**Table 1:** Primers sequences of hub genes and internal reference genes.

**Gene name**	**Primers sequences**	**Amplified fragment**
		**size**
*TSPAN1*	Forward 5':TGGGCTGCTATGGTGCTAAG	154 bp
	Reverse 5':GGCACTACCAGCAACGTCAG	
*TMPRSS4*	Forward 5':GGGAAGTCACCGAGAAGA	107 bp
	Reverse 5':ATGCCACTGGTCAGATTG	
*CTSE*	Forward 5':CTATACCCTCAGCCCAACTG	169 bp
	Reverse 5':GTTATTCCCACGGTCAAAGAC	
*SDR16C5*	Forward 5':AATGGGCTGGCAGATTACTG	111 bp
	Reverse 5':CACAATCGTGGTTTTGATCC	
*GAPDH*	Forward 5':TGACTTCAACAGCGACACCCA	121 bp
	Reverse 5':CACCCTGTTGCTGTAGCCAAA	

### Immunohistochemistry (IHC)

Specimens of 70 pancreatic cancer tissues and 70 adjacent tissues were deparaffinized and rehydrated. The sections were incubated with polyclonal anti-TSPAN1 antibody (1:1000 dilution) (SANTA CRUZ BIOTECHNOLOGY, sc-376551), anti-TMPRSS4 antibody (1:500 dilution) (proteintech, 11283-1-AP), anti-SDR16C5 antibody (1:300 dilution) (Thermo Fisher, PA5-55229), and anti-CTSE antibody (1:1000 dilution) (SANTA CRUZ BIOTECHNOLOGY, sc-166500). Two independent pathologists evaluated and scored the IHC, and the log2 (H-score) described the semi-quantitative expression of the four proteins.

### Diagnostic Model Development and Validation

In this analysis, the merged dataset was used to construct models of pancreatic cancer using four hub genes. A total of 818 samples were randomly assigned into a training cohort and a validation cohort at 7:3 ratios. The GSE32676 dataset was used as the external validation cohort. The support vector machine, random forest, Naive Bayes, neural network, linear discriminant analysis, mixture discriminant analysis, flexible discriminant analysis, and logistic regression were used to classify pancreatic cancer and normal tissues. To strengthen the robustness of the prediction with these genes, 10-fold cross-validation was also applied reiteratively 100 times. The receiver operating characteristic (ROC) curve was drawn to estimate the diagnostic performance of each model, and the sensitivity and specificity were determined. All statistical analyses were conducted using R 3.5.3.

## Results

### Gene Co-expression Network Construction and Key Modules Identification

**Figure 2:**
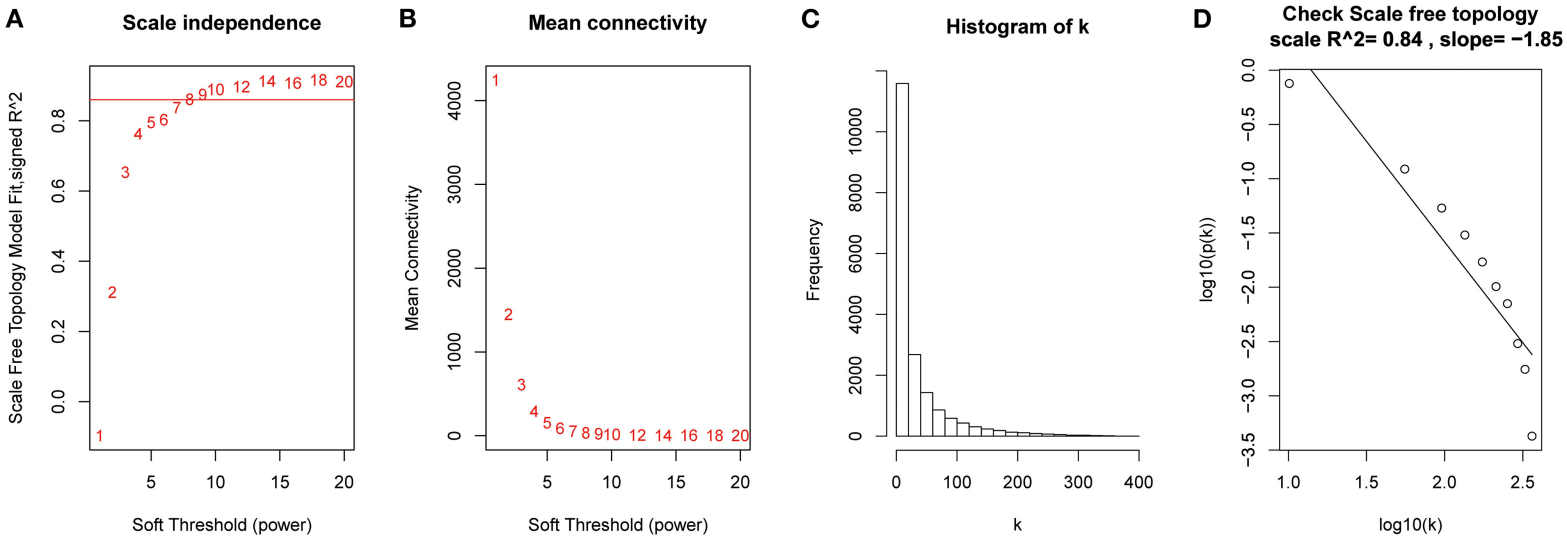
Determination of soft-thresholding power in the weighted gene co-expression network analysis (WGCNA). **(A)** Analysis of the scale-free fit index for various soft-thresholding powers (β). **(B)** Analysis of the mean connectivity for various soft-thresholding powers. **(C)** Histogram of connectivity distribution when β = 8. **(D)** Checking the scale-free topology when β = 8.

**Figure 3:**
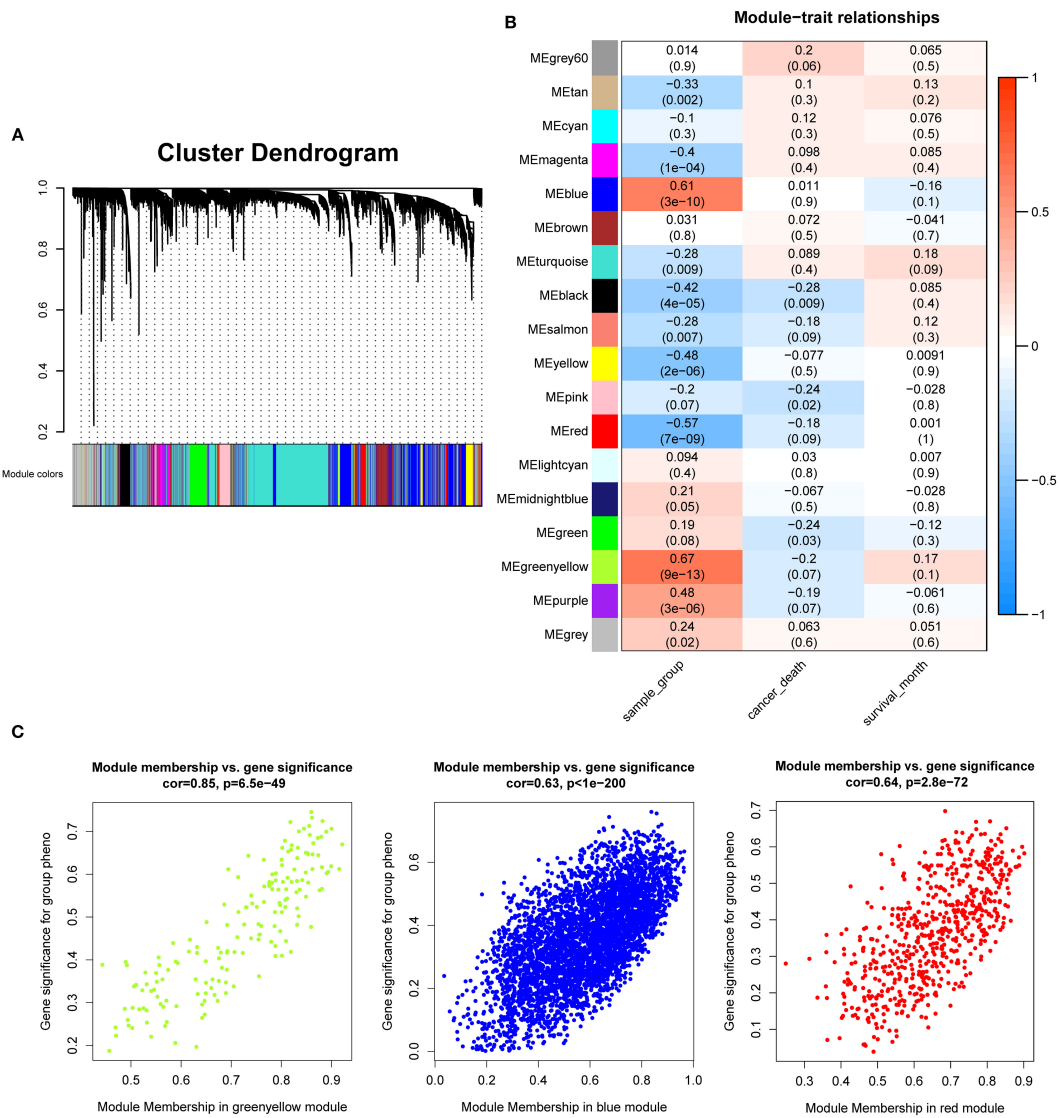
Identification of modules associated with the clinical traits of pancreatic cancer. **(A)** Dendrogram of 18,830 genes clustered based on a dissimilarity measure (1-TOM). **(B)** Heatmap of the correlation between module eigengenes and clinical traits of pancreatic cancer. **(C)** Module membership vs. gene significance in “greenyellow,” “blue,” and “red” module.

After the quality assessment for the GSE28735 dataset, GSM711915 and GSM711957 samples were removed. Eventually, a total of 18,830 genes and 88 samples were included to construct a gene co-expression network using the “WGCNA” package. In the current study, to ensure a scale-free network, β = 8 (scale-free R^2^ = 0.86) was selected (Figures 2A,B), and scale-free topology (R^2^ = 0.84, slope = −1.85) was obtained (Figures 2C,D). Through the obtained scale-free topology, 18,830 genes were classified as 18 modules (Figure 3A). Three modules were acquired that were significantly related to the sample category (greenyellow: *r* = 0.67, *P* = 9e-13; blue: *r* = 0.61, *P* = 3e-10; and red: *r* = −0.57, *P* = 7e-9; Figure 3B). The greenyellow module showed the highest correlation with clinical information (cor = 0.85, *P* = 6.5e−49, Figure 3C). Therefore, the 171 genes of the greenyellow module were used for subsequent analyses.

### Hub Gene Identification and Validation

**Figure 4:**
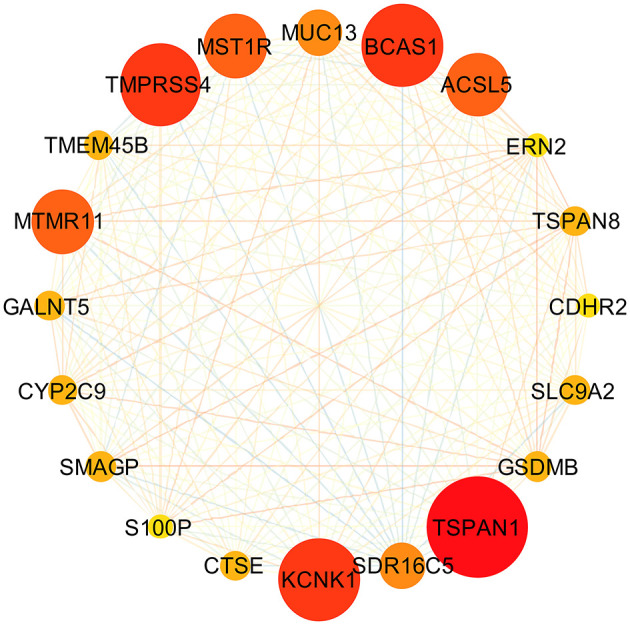
Gene-gene interaction network of the top 20 genes. Through constructing a gene-gene interaction network by using 171 genes obtained from WGCNA analysis, the top 20 genes, ranked by degrees of interactions, were identified.

**Figure 5:**
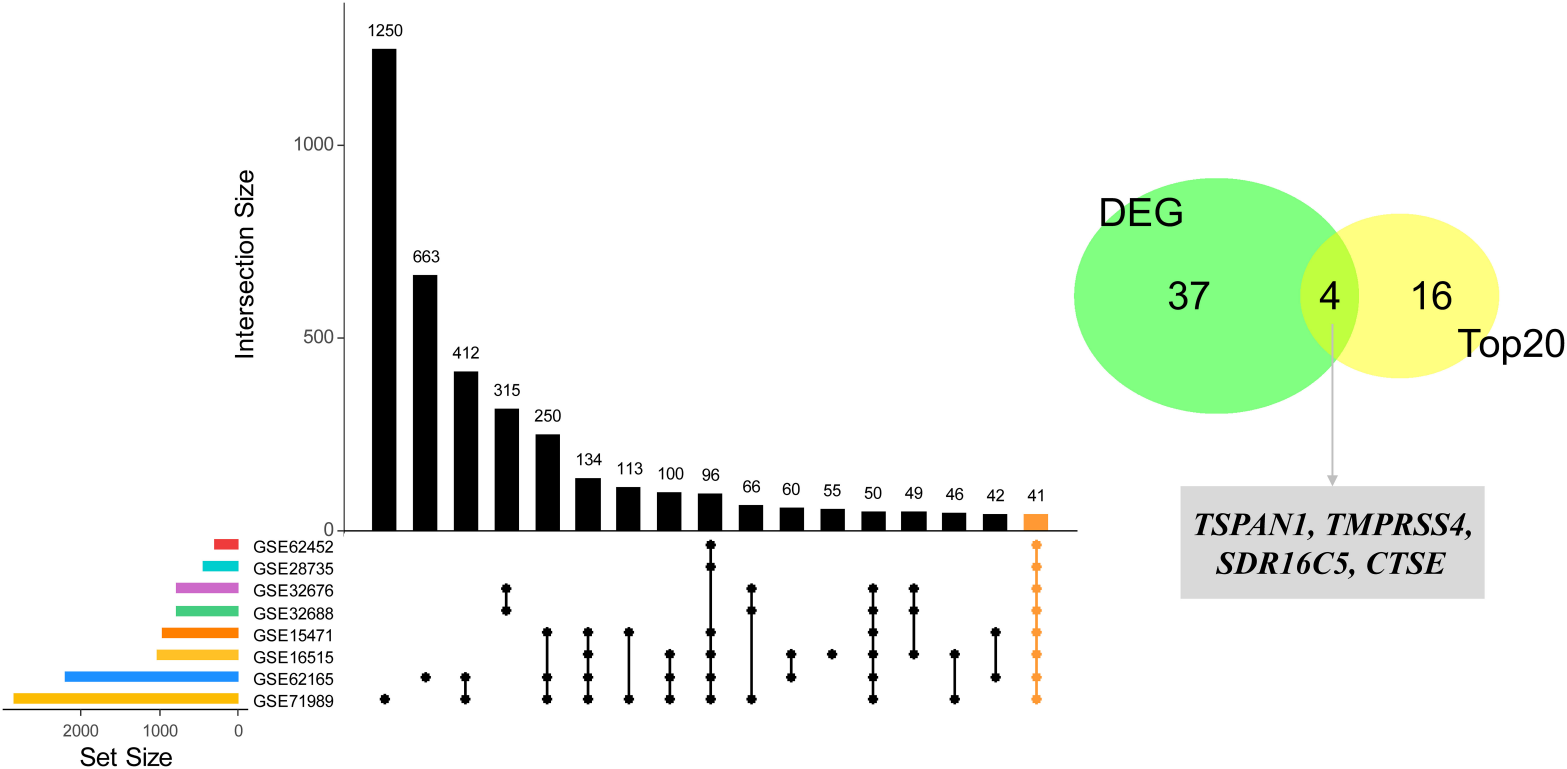
Identification of four hub genes by eight datasets validation. Forty-one DEGs were identified through the intersection of the DEGs of 8 GEO datasets (GSE15471, GSE28735, GSE62165, GSE32688, GSE71989, GSE62452, GSE62165, and GSE32676), and then four hub genes were identified by an intersection with the top 20 genes.

**Figure 6:**
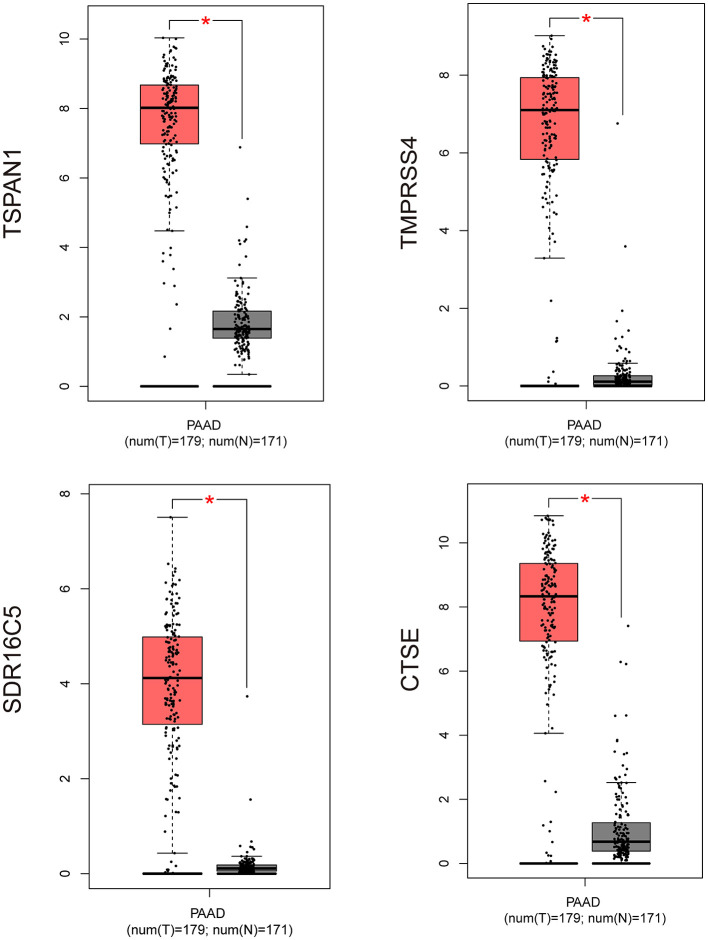
Validation of four hub genes expression by using RNA-Seq data (GEPIA website). *P ≤ 0.05; PAAD, pancreatic cancer (GEPIA website).

Based on the interaction parameters of the 171 genes obtained from WGCNA analysis, the top 20 genes were identified (Figure 4). DEGs analysis of eight gene datasets revealed a total of 41 genes, so four hub genes, *TSPAN1, TMPRSS4, SDR16C5*, and *CTSE* was identified (Figure 5). The validation results showed that four hub genes derived from the GEPIA tool were differentially expressed in cancer and normal tissues (Figure 6), as was the result of DESeq2 analysis (Supplemantary Table 2). The details of the four genes are shown in Table 2.

**Table 2:** Summary of four hub genes identified by weighted gene co-expression network analysis.

**Gene ID**	**Official full name**	**Description**	**References**
*TSPAN1*	Tetraspanin 1	Cell development, activation, growth, and motility	(31, 32)
*TMPRSS4*	Transmembrane serine protease 4	Integral component of membrane; regulation of gene expression; scavenger receptor activity	(33, 34)
*CTSE*	Cathepsin E	Antigen processing and presentation of exogenous peptide antigen via MHC class II; protein autoprocessing; protein catabolic process	(35, 36)
*SDR16C5*	Short chain dehydrogenase/reductase family 16C member 5	Activating transcription factor binding; keratinocyte proliferation; oxidation-reduction process	NA

### RT-PCR and IHC

**Figure 7:**
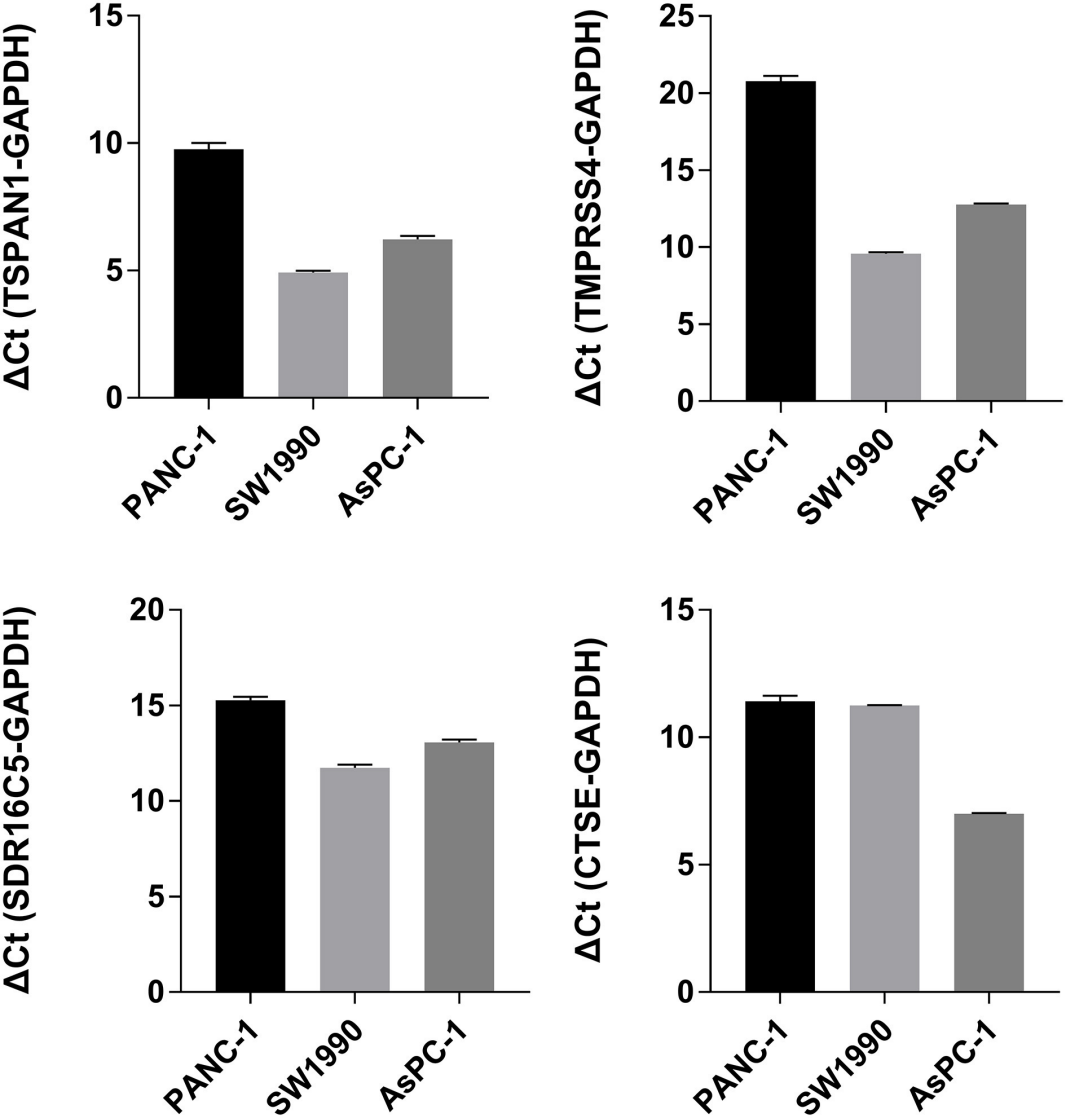
*TSPAN1, TMPRSS4, SDR16C5* and *CTSE* mRNA expression in three pancreatic cancer cells.

**Figure 8:**
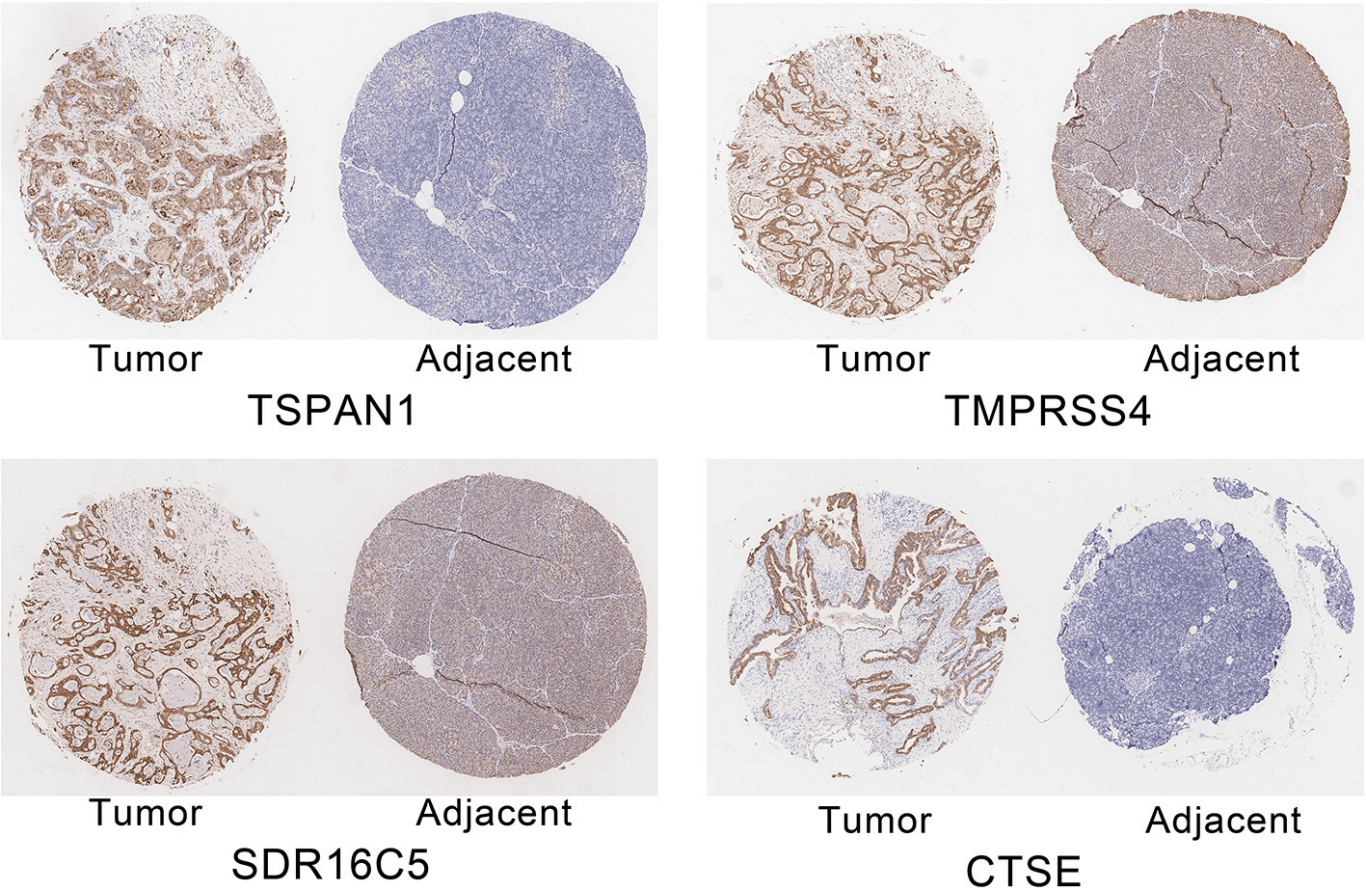
Immunohistochemical staining of TSPAN1, TMPRSS4, SDR16C5 and CTSE.

The expression of the four hub genes in three cell lines showed that *TSPAN1* and *CTSE* were expressed at high levels, *TMPRSS4* and *SDR16C5* were expressed at medium expression levels (Figure 7). IHC staining results are shown in Figure 8. The expression levels in pancreatic cancer tissues and adjacent tissues showed as follows: 7.27 ± 0.31 and 6.88 ± 0.14; 7.16 ± 0.24 and 7.02 ± 0.13; 7.15 ± 0.24 and 6.99 ± 0.14; 7.00 ± 0.26 and 6.76 ± 0.09. Higher levels of TSPAN1, TMPRSS4, SDR16C5 and CTSE expression were observed in pancreatic cancer than in normal pancreatic tissue (paired *t*-test, *P* < 0.0001).

### Diagnostic Model Development and Validation

In the validation cohort, the AUC of the eight models constructed by machine learning ranged from 0.87 to 0.92, sensitivity ranged from 0.91 to 0.94, and specificity ranged from 0.84 to 0.86. In the external validation cohort, the AUC of the eight models ranged from 0.86 to 0.98, sensitivity ranged from 0.84 to 1.00, and specificity ranged from 0.86 to 1.00 (Table 3).

**Table 3:** Diagnostic performance of eight machine learning methods for pancreatic cancer.

**Methods**	**Validation (30%)**			**External validation (GSE32676)**		
	**AUC**	**Se**	**Sp**	**AUC**	**Se**	**Sp**
Support vector machine	0.87 (0.79–0.95)	0.92	0.84	0.90 (0.73–1.00)	0.96	0.86
Random forest	0.91 (0.86–0.97)	0.91	0.86	0.94 (0.83–1.00)	0.96	0.86
Naive Bayes	0.91 (0.86–0.96)	0.93	0.84	0.92 (0.77–1.00)	0.96	0.86
Neural network	0.91 (0.86–0.97)	0.94	0.84	0.97 (0.91–1.00)	0.84	1.00
Linear discriminant analysis	0.91 (0.86–0.96)	0.93	0.84	0.95 (0.86–1.00)	1.00	0.86
Mixture discriminant analysis	0.91 (0.87–0.96)	0.91	0.84	0.98 (0.93–1.00)	1.00	0.86
Flexible discriminant analysis	0.91 (0.85–0.96)	0.92	0.84	0.86 (0.71–1.00)	0.84	0.86
Logistic regression	0.92 (0.87–0.97)	0.93	0.84	0.97 (0.90–1.00)	0.96	0.86

*AUC, receiver operating characteristic area under the curve value; Se, Sensitivity; Sp, Specificity*.

## Discussion

There is an urgent need for a relatively reliable, clinically easy to use, cost-effective biomarker panel for the diagnosis of pancreatic cancer. This study identified four hub genes through bioinformatics, DEGs analysis in multiple datasets, experimental verification of mRNA and protein levels. Using machine learning methods, the expression of four hub genes was utilized to construct models with satisfactory diagnostic value.

Pancreatic cancer is a polygenic and highly heterogeneous disease, the diagnosis of which is challenging (37). A single biomarker may not be sufficient for accurate diagnosis, and a panel consisting of multiple biomarkers might be more beneficial and accurate (38). In the study of pancreatic cancer, some diagnostic models have been developed (39–41). However, most models are not cost-effective for patients, because multiple biomarkers are difficult to routinely screen and/or identify clinically. Most importantly, a recent study demonstrated that a three-miRNA panel can be as effective as the panel of 1800 miRNAs (42). It is necessary to weigh the number of biomarkers in clinical application and their predictive abilities. Therefore, the focus of this study is to screen hub genes and explore a diagnostic model with cost-effective performance.

With the development of next-generation sequencing, bioinformatics has been used in many ways of research, such as biomarker screening, molecular mechanism exploration. Currently, WGCNA was widely applied to screen hub genes in various cancers (9). This approach can identify critical cancer driver genes that may be a significant therapeutic target or diagnostic marker (43). In recent years, several biomarkers have been identified in the field of cancer research using WGCNA (44–47). However, most studies only used DEGs or the first 25% variation genes to construct a weighted gene co-expression network, which may result in a loss of genetic diversity. Moreover, some studies only used the feature selection method to select biomarkers (17, 43, 48). Although this method can reduce the dimensionality of data, these genes that play important roles in the cancer process may be lost.

In this study, transcriptome data related to pancreatic cancer were systematically retrieved and its raw data were preprocessed. During the WGCNA analysis process, all genes were included in the construction of a co-expression network to find diagnostic biomarkers, which enhanced the diversity of genes. After using WGCNA to identify a set of genes highly correlated with pancreatic cancer, hub genes were identified through gene-gene interaction network analysis and DEGs analysis in independent eight datasets. It is important to emphasize the interactions between these genes, it can provide deeper insight into the mechanism of cancer (9, 49–51). To increase the credibility of the selected hub genes. DEGs validation was firstly applied using the RNA-Seq data. And then their gene and protein expression levels were verified through experimental methods, including RT-PCR and IHC methods.

In recent years, many studies have suggested that machine learning can provide promising tools for diagnosis in the cancer domain (13). For example, Pu et al. (52) identified a diagnostic model based on five hyper-methylated CpG sites with 0.82% accuracy using the support vector machine method. It is more practical to explore an optimal panel with few biomarkers and high diagnostic performance. Therefore, this study used the four hub gene expression profiles of 818 samples to construct the diagnostic models through machine learning. After internal verification and external verification, the results showed that panels of the four hub genes had a better diagnostic performance for pancreatic cancer.

Four hub genes were identified by bioinformatics in this study. *TSPAN1* (31, 32), *TMPRSS4* (33, 34), and *CTSE* (35, 36) have previously been studied in pancreatic cancer. Among them, *TMPRSS4* was overexpressed in, and identified as a biomarker of, pancreatic carcinoma (33), *TSPAN1, TMPRSS4*, and *CTSE* are potential diagnostic or prognostic markers for pancreatic ductal adenocarcinoma (31, 33, 35), and most of these genes are associated with metastasis and proliferation and in pancreatic cancer. Although *SDR16C5* has not been reported in pancreatic cancer, a study showed that it is involved in the regulation of triple-negative breast cancer (53). Its potential as diagnostic marker warrants further functional investigations on its roles in the development of pancreatic cancer.

Certain important strengths of this study should be emphasized. First, the data used in this study are very comprehensive, and the sample size is the largest in the current study of pancreatic cancer. Second, multiple validations of hub genes expression were executed using eight microarray data sets and RNA-Seq data sets, and the RT-PCR and IHC methods were used to validate their expression at the gene and protein level. Those validations can maximize the reliability of the selected hub genes. Third, logistic regression and several machine learning methods were applied to evaluate the diagnostic ability of our panels. Iterative ten-fold cross-validation repeated 100 times was also used to obtain a robust evaluation of the prediction ability using these genes. There are also some limitations in this study. First, the research samples included in this study were from diverse populations from the USA, France, and Japan. There may exist some differences in gene expression profiles among various ethnic groups. Next, our prediction models will be improved with further validation using independent experimental data.

In conclusion, four hub genes were identified using bioinformatics and experimental verification approaches. More importantly, the four-gene panels can accurately predict pancreatic cancer. Our findings encourage future clinical research to validate the robustness of the diagnostic model and additional functional research.

## Data Availability Statement

The original contributions presented in the study are included in the article/Supplementary Material, further inquiries can be directed to the corresponding author/s.

## Ethics Statement

The studies involving human participants were reviewed and approved by Life Science Ethics Review Committee of Zhengzhou University. The patients/participants provided their written informed consent to participate in this study.

## Author Contributions

TL conceived the project. HW, JW, and CY collected the datasets. TL participated in the pre-processing of the datasets and performed the computational analysis. TL and HY drafted the manuscript. JS, PW, CS, LD, GJ, YH, JL, and YY thoroughly revised the manuscript. All authors read and approved the final manuscript.

## Conflict of Interest

The authors declare that the research was conducted in the absence of any commercial or financial relationships that could be construed as a potential conflict of interest.

## Abbreviations

WGCNA: Weighted gene co-expression network analysis
DEGs: differentially expressed genes
TOM: topological overlap matrix
GS: gene significance
MM: module membership
TCGA: the Cancer Genome Atlas
ICGC: International Cancer Genome Consortium
GTEx: Genotype-Tissue Expression
ROC: receiver operating characteristic
Se: Sensitivity
Sp: Specificity.
