# Supplementary material for: TSPAN1, TMPRSS4, SDR16C5, and CTSE as Novel Panel for Pancreatic Cancer: A Bioinformatics Analysis and Experiments Validation
Source: Front Immunol. 2021 Mar 18;12:649551. doi: 10.3389/fimmu.2021.649551 (PMC8015801; doi:10.3389/fimmu.2021.649551)
Supplement: Supplementary file 1 [file Presentation_1.zip › Supplementary Material.docx]

# Supplementary Material

**Figure S1** PCA chart before and after batch effect correction using the “sva” package.

**Table S1**. Summary of 11 datasets used in this study.

**Table S2**. Differential analysis of four hub genes using the “DESeq2” package.

**R code** R language code for data processing, WGCNA, and machine learning, etc.

**Datasets** gse28735_WGCNA: the expression profile of GSE28735; nineGeo_4hub: microarray of model development; gse32676_valiData: microarray of model external validation;
