# Supplementary material for: TSPAN1, TMPRSS4, SDR16C5, and CTSE as Novel Panel for Pancreatic Cancer: A Bioinformatics Analysis and Experiments Validation
Source: Front Immunol. 2021 Mar 18;12:649551. doi: 10.3389/fimmu.2021.649551 (PMC8015801; doi:10.3389/fimmu.2021.649551)
Supplement: Supplementary file 1 [file Presentation_1.zip › Table S1.pdf]

Table S1 Summary of 11 microarray datasets used in this study

Accession	Platform full name	Platform abbreviation	Samples (tumor/normal)	Database source	Region	Role in this study
GSE28735	[HuGene-1_0-st] Affymetrix Human Gene 1.0 ST Array	GPL6244	90 (45/45)	GEO	USA	WGCNA
E-MEXP-2780	[HG-U133_Plus_2] Affymetrix Human Genome U133 Plus 2.0 Array	GPL570	30 (30/0)	ArrayExpress	Germany	Model Development/Validation
GSE15471	[HG-U133_Plus_2] Affymetrix Human Genome U133 Plus 2.0 Array	GPL570	78 (39/39)	GEO	Romania	Model Development/Validation
GSE16515	[HG-U133_Plus_2] Affymetrix Human Genome U133 Plus 2.0 Array	GPL570	52 (36/16)	GEO	USA	Model Development/Validation
GSE32688	[HG-U133_Plus_2] Affymetrix Human Genome U133 Plus 2.0 Array	GPL570	32 (25/7)	GEO	USA	Model Development/Validation
GSE71989	[HG-U133_Plus_2] Affymetrix Human Genome U133 Plus 2.0 Array	GPL570	21 (13/8)[a]	GEO	USA	Model Development/Validation
GSE106189	[HG-U133_Plus_2] Affymetrix Human Genome U133 Plus 2.0 Array	GPL570	35 (35/0)	GEO	Japan	Model Development/Validation
GSE62452	[HuGene-1_0-st] Affymetrix Human Gene 1.0 ST Array	GPL6244	130 (69/61)	GEO	USA	Model Development/Validation
E-MTAB-6134	[HG-U219] Affymetrix Human Genome U219 Array	GPL13667	309 (309/0)	ArrayExpress	France, Belgium	Model Development/Validation
GSE62165	[HG-U219] Affymetrix Human Genome U219 Array	GPL13667	131 (118/13)	GEO	Belgium	Model Development/Validation
GSE32676	[HG-U133_Plus_2] Affymetrix Human Genome U133 Plus 2.0 Array	GPL570	32 (25/7)	GEO	USA	Model External Validation

Note: [a]The GSM1849348 sample was removed from GSE71989 dataset because the patient is a sample of pancreatitis. The data sets of GPL570, GPL6244 and GPL13667 are annotated by “hgu133plus2.db”, “hugene10sttranscriptcluster.db”, “hgu219.db” packages respectively. If one gene was detected by multiple probes, the max value was used to represent the expression level.
