# Supplementary material for: TSPAN1, TMPRSS4, SDR16C5, and CTSE as Novel Panel for Pancreatic Cancer: A Bioinformatics Analysis and Experiments Validation
Source: Front Immunol. 2021 Mar 18;12:649551. doi: 10.3389/fimmu.2021.649551 (PMC8015801; doi:10.3389/fimmu.2021.649551)
Supplement: Supplementary file 1 [file Presentation_1.zip › Table S2.pdf]

Table S2 Differential analysis of four hub genes using the "DESeq2" package

Gene symbol	baseMean	log2FoldChange	lfcSE	stat	p-value	p adj
TSPAN1	4179.71	5.41	0.09	59.41	0.00*	0.00*
TMPRSS4	4045.19	8.59	0.15	55.40	0.00*	0.00*
SDR16C5	658.12	6.27	0.12	53.30	0.00*	0.00*
CTSE	11698.18	6.53	0.14	45.52	0.00*	0.00*

*: $P<0.05$
