# Supplementary figures and images for: TSPAN1, TMPRSS4, SDR16C5, and CTSE as Novel Panel for Pancreatic Cancer: A Bioinformatics Analysis and Experiments Validation
Source: Front Immunol. 2021 Mar 18;12:649551. doi: 10.3389/fimmu.2021.649551 (PMC8015801; doi:10.3389/fimmu.2021.649551)

A

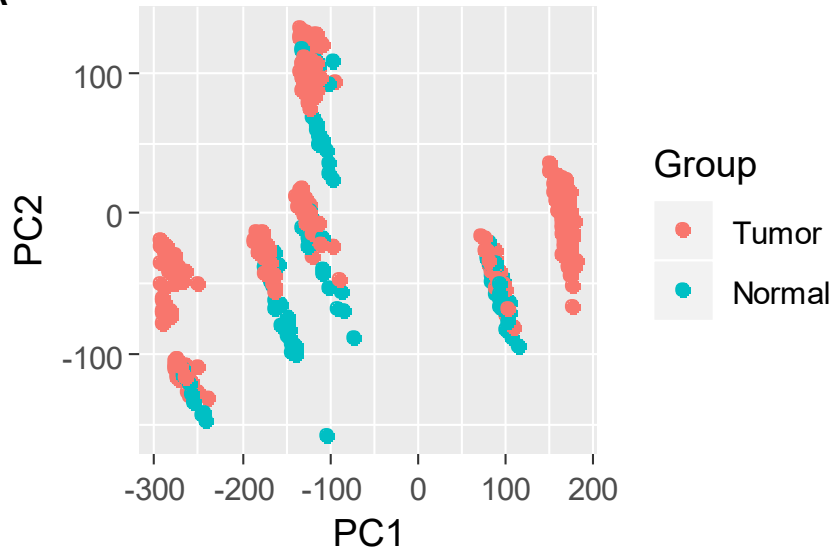

B

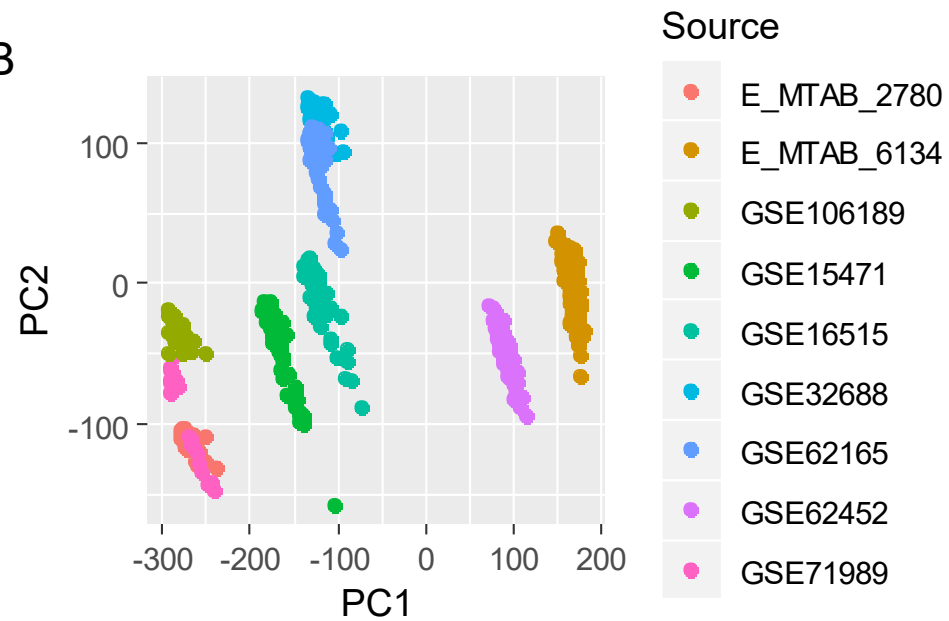

C

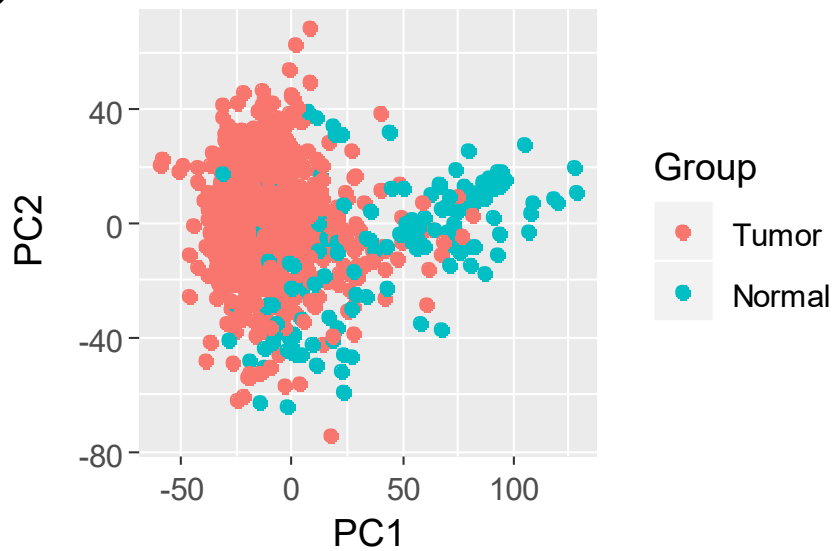

D

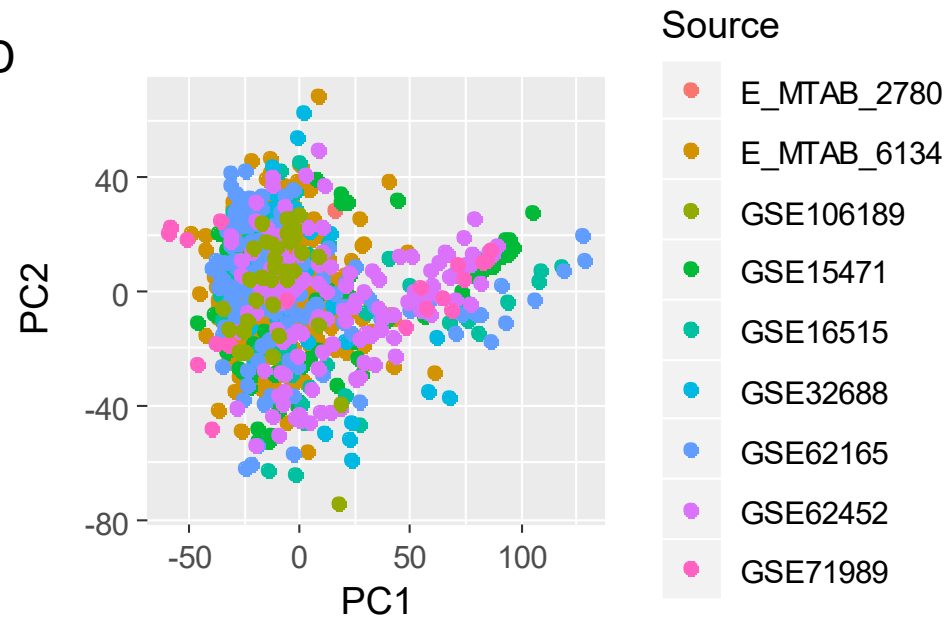

Supplement: Supplementary file 1 [file Presentation_1.zip › Figure S1.pdf]
